# Cost-effectiveness of a combined intervention of long lasting insecticidal nets and indoor residual spraying compared with each intervention alone for malaria prevention in Ethiopia

**DOI:** 10.1186/s12962-018-0164-1

**Published:** 2018-11-22

**Authors:** Alemayehu Hailu, Bernt Lindtjørn, Wakgari Deressa, Taye Gari, Eskindir Loha, Bjarne Robberstad

**Affiliations:** 1 Department of Global Public Health and Primary Care, Centre for International Health, University of Bergen, Bergen, Norway; 2 Department of Reproductive Health and Health Service Management, School of Public Health, Addis Ababa University, Addis Ababa, Ethiopia; 3 Department of Preventive Medicine, School of Public Health, Addis Ababa University, Addis Ababa, Ethiopia; 4 School of Public and Environmental Health, Hawassa University, Hawassa, Ethiopia; 5 Center for Intervention Science in Maternal and Child Health (CISMAC), University of Bergen, Bergen, Norway

**Keywords:** Malaria, Malaria prevention, Economic evaluation, LLIN, IRS, Cost-effectiveness, Ethiopia

## Abstract

**Background:**

The effectiveness of long lasting insecticidal nets (LLINs) and indoor residual spraying (IRS), for malaria prevention, have been established in several studies. However, the available evidence about the additional resources required for a combined implementation (LLIN + IRS) with respect to the added protection afforded is limited. Therefore, the aim of this study was to compare the cost-effectiveness of combined implementation of LLINs and IRS, compared with LLINs alone, IRS alone, and routine practice in Ethiopia.

**Methods:**

The study was performed alongside a cluster randomized controlled trial of malaria prevention conducted in Adami Tullu district, in Ethiopia, from 2014 to 2016. In addition, literature-based cost-effectiveness analysis—using effectiveness information from a systematic review of published articles was conducted. Costing of the interventions were done from the providers’ perspective. The health-effect was measured using disability adjusted life years (DALYs) averted, and combined with cost information using a Markov life-cycle model. In the base-case analysis, health-effects were based on the current trial, and in addition, a scenario analysis was performed based on a literature survey.

**Results:**

The current trial-based analysis showed that routine practice is not less effective and therefore dominates both the combined intervention and singleton intervention due to lower costs. The literature-based analysis had shown that combined intervention had an incremental cost-effectiveness ratio of USD 1403 per DALY averted, and USD 207 per DALY averted was estimated for LLIN alone. In order for the ICER for the combined intervention to be within a range of 1 GDP per capita per DALY averted, the annual malaria incidence in the area should be at least 13%, and the protective-effectiveness of combined implementation should be at least 53%.

**Conclusions:**

Based on the current trial-based analysis, LLINs and IRS are not cost-effective compared to routine practice. However, based on the literature-based analysis, LLIN alone is likely to be cost-effective compared to 3 times GDP per capita per DALY averted. The annual malaria probability and protective-effectiveness of combined intervention are key determinants of the cost-effectiveness of the interventions.

*Trial registration* PACTR201411000882128 (Registered 8 September 2014). http://www.pactr.org/ATMWeb/appmanager/atm/atmregistry?dar=true&tNo=PACTR201411000882128

## Background

Scale up of malaria prevention—mainly with the mass distribution of long lasting insecticidal nets (LLINs) and indoor residual spraying (IRS) of the interior lining of the wall of the houses—have brought a remarkable reduction in the global burden of malaria in the last decade [1, 2]. Empirically, the effectiveness and the cost-effectiveness of both LLINs and IRS, for malaria prevention, are well-established [3–7]. However, evidence also indicates that neither LLIN nor IRS—alone—will be sufficient to reach and maintain the interruption of transmission in highly malarious regions of Africa [8–10].

In Ethiopia, LLINs and IRS are usually implemented separately in different districts or different villages [11, 12]. While LLINs and IRS in some villages have been implemented simultaneously within the same households [12–16], little is known about the effect and cost-effectiveness of the combined use of LLINs and IRS [12, 17]. Moreover, are the additional costs of the combined interventions reasonable from a provider’s perspective given the combined benefits? [7].

Mathematical models by Yakob et al. [18], Okumu et al. [19], and Chitnis et al. [20] shows that there might be some additional protective value by a combined implementation of LLINs and IRS compared to either of them alone. A review of cross-sectional data from 17 different countries in sub-Saharan Africa also shows that people in households which use both bed-nets and IRS are about 36% (95% CI 7%–53%) better protected compared to households which only use one of the interventions in medium malaria transmission areas [16]. Similarly, studies from Kenya [21] and Tanzania [22] also report positive results of combining LLINs and IRS. Kleinschmidt et al. based on literature search and a cross-sectional survey from Bioko island of Equatorial Guinea, conclude that the increased resource use of the combined intervention is justifiable because of additional effectiveness compared with each intervention alone [23]. On the other hand, randomized trials from Benin [24], Gambia [25], and Sudan [26] report that there is no added effect in the combined implementation, compared with each intervention implemented separately.

However, none of those studies estimated the effect at a general population level, nor did they attempt to evaluate the cost and cost-effectiveness of the interventions. In the battle against malaria the need for transparent evidence based on randomized controlled trials, which integrates robust decision modelling, is critical to allocate scarce resources appropriately [27]. Such evidence will be useful to guide the selection of the packages of interventions for malaria elimination programs. Specifically, the pressing questions in this line of inquiry are; first, what are the additional effects of combining both LLIN and IRS compared with singleton interventions or the routine practice? Second, is the value of added protection substantial enough to justify the additional resources (i.e. cost) required for a combined implementation? Therefore, the aim of this study was to compare the cost-effectiveness of combined implementation of IRS and LLIN, compared with LLIN alone, IRS alone and routine practice in Ethiopia.

## Methods

### Study design and settings

This cost-effectiveness study was conducted alongside a cluster randomized controlled trial of malaria prevention, with a 2 × 2 factorial design (*MalTrials*), which compared both the effectiveness, cost, and cost-effectiveness of combined implementation of universal coverage of Long Lasting Insecticidal Nets and Indoor Residual Spraying (LLIN + IRS) against universal coverage of LLIN alone, IRS alone, and the routine practice [28]. *MalTrials* also has substantial entomological components that compare vector outcomes. Furthermore, to improve external validity, this study considers the cost-effectiveness under a scenario of varying annual malaria incidence and different levels of protective-effectiveness of the interventions based on a literature survey.

The trial was conducted in 2014–2016 in Adami Tullu (*Adami Tullu Judo Kombolcha*) district, which has a population of about 170 thousand [29]. Adami Tullu is located in the heart of the Great Rift Valley. The elevation of the district ranges from about 1500 to 2300 meters above sea level, with most of the inhabited villages located in the lower parts. The annual mean temperature ranges from a minimum of 14 °C to a maximum of 27 °C. Like most places in Ethiopia, the district has two rainy seasons, the longer (June to September) and the shorter (February to April). However, the rainfall patterns are irregular and this contributes to the variability of malaria incidence in the area. Malaria is also one of the top causes of outpatient visits and inpatient admission in Adami Tullu and surrounding area [30]. The pilot study for this trial showed an annual malaria incidence of 24 per 1000 population at risk in this area [31].

### Description of the interventions compared

The detail descriptions of the interventions and the size of sample population are provided in the published protocol [28]. In brief, 44 clusters were included in each study arm, and a total of 6071 the universal coverage of households with Long Lasting Insecticidal Nets entailed each household receiving free LLINs (*PermaNet 2.0*) in October 2014. The distribution of the LLIN was conducted based on the national malaria guideline [32], which recommends proportional allocation of bed nets to the size of the household. Health extension workers distributed the bed net from health posts. A day before the distribution date, all households in these groups were mobilized by the administration of the villages (i.e. the ‘chair-person’ and the manager) to come to the health posts for collecting the bed nets, after which the LLIN coverage was 99%.

The second intervention was universal coverage of households with indoor residual spraying the insecticide *Propoxur* (isopropoxy-phenyl methylcarbamate), which is currently considered effective. The spraying was conducted annually in September 2014, July 2015, and July 2016. On average, the IRS coverage was about 95% for each of the three rounds of spraying. We followed the national indoor residual spraying operation guideline [32]. A 6-day training on spraying operation was given for locally recruited 13 spray teams and 4 supervisors. The personnel were organized in a teams of four spray-men, a porter, and squad leaders (health extension workers). The four supervisors were malaria focal persons from the district health office. In addition, a community sensitization was performed to inform residents about safety, purpose, and time of spraying. Nearly 12 houses were sprayed by each spray-team per day using a spray pump which has an 8-l capacity.

In the combined implementation arms, households received LLINs and IRS in parallel with households in the individual arms, and therefore had IRS coverage of 95% and LLIN coverages of 99%. Finally, in the routine arm, neither LLIN distribution nor IRS was implemented by either the study project or by the district health office within the study period, and the background coverage of LLINs and IRS based on the baseline survey was on average 11%. Table 1 summarises the description of the interventions and participant population in each of the four arms of the trial.

**Table 1:** Description of the interventions, combinations of intervention and routine arms

Study arms	Number of households	Description of the interventions
LLIN alone	1387	Universal coverage of households with LLINs: each household received free LLINs (*PermaNet 2.0*)—proportional allocation to the household size —99% coverage immediately after distribution (October 2014)
IRS alone	1526	Universal coverage of households with IRS: using *Propoxur* (isopropoxy-phenyl methylcarbamate) each house sprayed once every year—about 95% coverage for each of the three rounds of spraying (September 2014, July 2015, and July 2016)
Combination (LLIN + IRS)	1615	Each household received LLINs and IRS in parallel with households in the individual arms, and therefore had IRS coverage of 95% and LLIN coverages of 99%
Routine	1541	Neither LLIN, nor IRS was implemented by either the study project or by the district health office within the study period. Based on the baseline data, the background coverage of LLINs was 11%

### Cost-effectiveness modelling

**Fig. 1:**
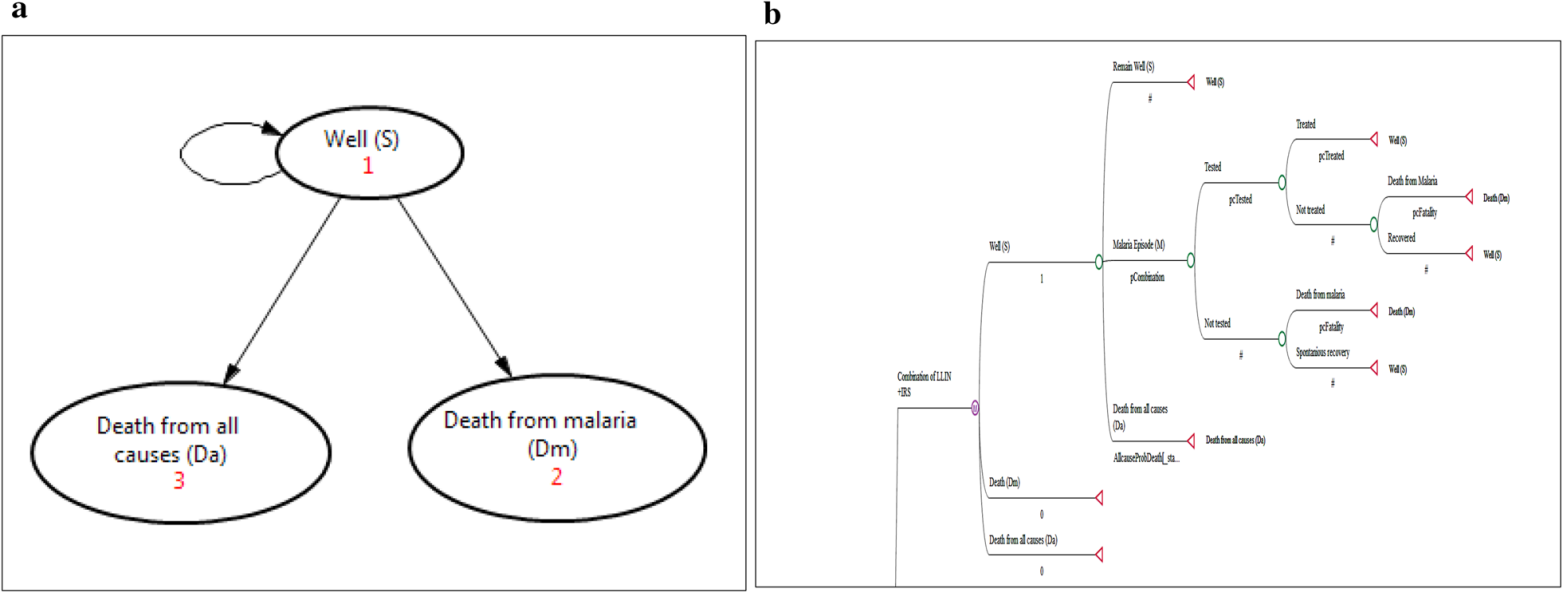
**a** Markov state-transitions diagram, and **b** Markov tree diagram for the model

We developed a simple malaria transmission model (Fig. 1) and populated it with effectiveness and cost data from the trial. We used TreeAge Pro Suit 2017 (© 2017 TreeAge Software, Inc.) software for building the model and for data analysis. Three mutually exclusive health states that represent the dynamics of malaria were defined: well (S), death from malaria (Dm), and death from all other causes (Da). According to this model, initially, all individuals are in ‘well’ (S) state, and they all are susceptible to malaria. Then, a person from a ‘well’ state (S) could be infected and experience a malaria episode (M) with a certain probability. Once inflicted with malaria (M), some could be diagnosed, treated, and cured; while some might not be diagnosed and therefore remain untreated (Fig. 1). In order to account for ongoing risk, recurrent nature, and short duration of malaria illness, we consider malaria episode as ‘temporary states’ [33].

In this model, we followed a hypothetical Ethiopian birth cohort over their lifetime (i.e. the time horizon in this evaluation was 80 years). A similar Markov life-cycle cohort model was employed for each intervention group (LLIN alone, IRS alone, LLIN + IRS) and control group (Routine). Each state was associated with annual state rewards, related to spending a year in the particular health state. These include the annual cost of prevention and the annual effectiveness value: DALY averted. Health systems cost of malaria diagnosis and treatment, and *dis*-*utility* from malaria episode were accounted as a transition rewards per event. Both the cost and health effect were non-differentially discounted with 3% discount rate.

Transition probabilities were used to capture the probabilities of moving from one state to another state—within a specific time period called cycle length. The cycle length in this model is defined as 1 year. A half-cycle correction was done in order to assume that events occur half-way through a cycle (rather than at the beginning or at the end). We base the transition probabilities on primary data (i.e. based on the incidence data from the trial) and a few estimates from the World Health Organization (WHO) (Table 2). The most likely annual probabilities for malaria were computed from the trial result of malaria incidence per 1000 persons year of observation (PYO) which were 15.548 for combination, 15.184 for LLINs alone, 15.652 for IRS alone, and 15.144 for routine arms. Then, we applied a formula, P = 1 − e^−rt^, in order to convert the incidences into transition probabilities using, where P is probability, e base of natural logarithm, r is incidence rate, and t is time period [34]. The model includes age-specific all-cause mortality rates (Da) from WHO population life-table [35], and malaria-specific death rate (Dm) (i.e. 1 per 100 untreated cases) from WHO estimate [36].

**Table 2:** Probabilities and costs (2014 USD) used in cost-effectiveness analysis of combined intervention of LLIN and IRS

Parameters^a^	Most likely	Min.	Max.	SD	Source
Probability of malaria in combined arm	0.0154	0.0146	0.0162	0.0004	Primary
Probability of malaria in LLIN arm	0.0151	0.0143	0.0159	0.0004	Primary
Probability of malaria in IRS arm	0.0155	0.0147	0.0163	0.0004	Primary
Probability of malaria in routine arm	0.0140	0.0133	0.0147	0.0004	Primary
Proportions of malaria cases tested (%)	90	80	100	0.0500	Primary
Proportions of malaria cases treated (%)	90	80	100	0.0500	Primary
Probability of death from untreated malaria	0.01	0.005	0.02	0.0004	[36]
Intervention cost of LLIN + IRS	4.04	3.00	4.500	0.2020	Primary
Intervention cost of LLIN	1.06	0.848	1.272	0.0530	Primary
Intervention cost of IRS	3.07	2.456	3.684	0.1535	Primary
Intervention cost of routine	0	0	0	0.0000	Primary
Cost of malaria diagnosis at PHCU	0.51	0.408	0.612	0.0255	Primary
Cost of malaria treatment at PHCU	1.17	0.936	1.404	0.0585	Primary
DALY: disability weight-malaria	0.191	0.172	0.211	0.0098	[38]
DALY: disability weight-death	1	1	1		[38]
DALY: disability weight-well	0	0	0		[38]
Discount rate health utility (%)	3	0	5		[40]
Discount rate cost (%)	3	0	5		[40]
Number of cycles (year)	80	10	80		[43]

*SD* standard deviation, *Min* minimum value, *Max* maximum value, *GBD* Global Burden of Disease study

^a^The values for Probabilities and DALYs are presented per cycle while Costs and Proportions are presented per event

### Measurement of health effect

The health effect for the trial-based cost-effectiveness analysis was entirely based on the randomized controlled trial results (*Maltrials*). We later relaxed this presumption to perform a scenario analysis (literature-based cost-effectiveness analysis) on expected values of incidence and effectiveness from literature survey. Using the malaria incidence information, disability adjusted life years (DALYs) averted was used as a health outcome measure in this analysis. The DALYs estimate combines the years of life lost (YLL) due to premature death and years of life lived with disability (YLD) [37]. The YLDs for malaria infection was calculated using standard disability weights (Table 2) [38]. Since a DALY is a negative measure (which we aim to reduce), we inverted the incremental effects when presenting cost-effectiveness results to get correct ranking of the alternatives. Death due to treated uncomplicated malaria is very rare and assume zero mortality. Uncomplicated malaria might progress to severe malaria if untreated, and we, therefore, assume a mortality of 1 per 100 for untreated cases [36].

In order to estimate malaria incidence, both active and passive malaria case detection methods were implemented intensively in four study arms. Every household was visited every week and asked if there was any household member who had a fever in the last 48 h. All febrile cases were then tested with a rapid diagnostic test (RDT) and blood slides were collected for confirmatory diagnosis.

### Measurement of intervention costs

Identification, measurement, and valuation of the cost of the intervention and cost of malaria diagnosis and treatment was conducted from providers’ perspective [27]. The costing of prevention interventions was conducted along-side the implementation of indoor residual spraying and the distribution of LLINs using ingredient costing approach.

#### Identification

All costs related to the undertaking or facilitation of the research activities were excluded. For LLINs, the purchasing cost of the bed nets (LLINs), shipment, customs clearance, and transportation fee to the project implementation district were included in the analysis. Moreover, at the project site, cost of transportation including payments for loading and unloading of the nets, rent fee for storage space, stationary materials for orientation training and data registration cost were included. On the dates of distribution, personnel cost and transportation cost of the bed nets to each of the villages were included. For the IRS, cost of the insecticide (*Propoxur*), spraying materials, equipment, storage, personnel, and other operating expenditures used for the indoor residual spraying were accounted.

#### Measurement

Cost data were collected prospectively, immediately starting from the beginning of the trial using financial expenditure records (invoices) from the project accountant services of the implementation of the interventions and from the district health office. We used a spreadsheet to record cost information. The types and quantity of each resource used in the intervention were registered.

We captured the economic costs of the interventions, whether they incurred a financial expenditure or not. For example, the time spent by health personnel involved in prevention or treating malaria was accounted, despite that their salaries were already covered by health services. While they did not receive additional salaries for the specific malaria intervention being evaluated in the trial, they could have spent their time on other activities representing opportunity costs.

#### Valuation

In order to identify the economic value of the resources used, we used the purchasing price for most of the materials and equipment, including for the bed nets and the insecticides. For items where the price was not known from the invoice or the available records, we use estimated values for the items from market inventory data. Cost items were divided into recurrent and capital costs. Recurrent costs were defined as costs which are incurred regularly and with duration of less than a year. Capital costs were defined as items, expected to last longer than 1 year [27]. Capital costs were annuitized based on the useful life-year, initial unit price, and interest rate of 6% [39]. For example, LLIN costs were assumed to be effective for 2 years, and hence the purchase cost was annualized over this period.

We valued personnel cost based on an estimated proportion of working-time spent for malaria prevention intervention for permanently employed staff. The health center heads, malaria focal persons, district health office head, district health office deputy head, zonal health desk malaria coordinator, store keepers, casher, and guard spent about 10% of their time for the IRS or LLINs distribution during the intervention days; while the health extension workers, health centre heads, and malaria focal persons spent about 50% of their time for the interventions. This information were used as allocation factors for the per-dime and salary rates. However, the spray-men and porters, who were temporary staffs and fully dedicated for the interventions during the implementation period, all per diem and salary costs were allocated to the interventions.

Unit costs were calculated by dividing the total cost of the intervention for the total population covered with the specific interventions. All costs were converted to USD using the official National Bank of Ethiopia average exchange rate for 2014 (USD 1 = ETB 20.1). We used a consumer price index in order to account for annual inflation. The reference year for all cost estimates in this study is 2014 USD.

For costs in the routine arms, we only accounted the cost of case diagnosis and treatment of malaria in the health facilities (health posts and health centre). We used Microsoft Excel (2016) for compilation and analysis of the cost data.

### Measurement of diagnosis and treatment cost of malaria

A combination of top-down and activity-based costing techniques was applied in order to track all cost items. Using Excel spreadsheet, we systematically extract data on expenses for testing and treating a case of malaria from the providers’ perspective. Primary cost data on diagnosis and treatment of malaria were collected from the same district where the trial was conducted, but from Primary Health Care Units (PHCU) which were not included in the study area. The data was collected from 9 Health Posts, 3 Health Centres, the District Health Office (Adami Tullu), Oromia Regional Health Bureau, and Federal Ministry of Health Pharmaceuticals Funds and Supply Agency (PFSA).

Personnel cost includes the cost of health professionals’ time involved in treating malaria. The average time spent on diagnosis and treatment of a case of malaria was combined with the apportioned net monetary value of the personnel time to estimate the personnel cost. At Health Centres, Health Officers, Nurses, Laboratory Technicians, and other administrative staffs were involved in the diagnosis and treatment of malaria cases, while only the Health Extension Workers were involved at Health Posts. We divided the entire treatment process into a set of activities along the clinical pathway and allocated monetary values for the drug and other supplies consumed for each activity. Finally, all cost were converted to 2014 USD.

### Cost-effectiveness analysis (CEA)

Incremental cost-effectiveness ratio (ICER), cost-effectiveness scatterplot, and cost-effectiveness acceptability curve were used to summarize and present the cost-effectiveness result [27]. The expected costs and health outcomes (DALY averted) were calculated for each of the four alternative options. We ranked all interventions in ascending order in terms of cost of interventions, and each intervention was therefore compared with the next costly intervention to calculate the incremental costs, the incremental effectiveness, and the incremental cost-effectiveness ratio (ICER). We eliminated from comparison the interventions that costed more but provided fewer benefits than an alternative intervention (dominance).

Based on the economic theory of maximization of expected health benefits from the interventions, the optimal decision is to choose the strategy with the highest ICER per DALY that just falls at or below the willingness to pay (WTP) threshold [40]. In this study we applied the WTP threshold suggested by World Health Organization’s (WHO’s) Choosing Interventions that are Cost-Effective (CHOICE) program that interventions for which the ICER per DALY averted is less than one GDP per capita as ‘very cost-effective’, between one and three-times GDP per capita as ‘cost-effective’, and greater than three-times GDP per capita as ‘not cost-effective [41]. The GDP per capita in Ethiopia for the year 2014 was USD 628 [42].

### Probabilistic sensitivity analysis

We analysed overall-model uncertainty with probabilistic sensitivity analyses (PSA) using Monte Carlo simulation, and the results are presented as cost-effectiveness acceptability curves, cost-effectiveness acceptability frontiers, and scatter plots. In the PSA the variables in the model were replaced with distributions. Probabilistic distributions for costs, dis-utilities, and transition probabilities were assigned with most likely (mean), minimum, and maximum values. We assumed cost parameters to hold gamma distributions, and health outcome and transition probabilities to follow beta distributions. We considered the minimum and maximum transition probabilities to vary ± 5% from the most likely values, and the minimum and maximum intervention costs to vary ± 20% from the most likely values (Table 2).

## Scenario analysis with literature-based cost-effectiveness model

The overall incidence of malaria in the study area was low during the study period compared with historical data and national average estimate [44]. Even though the interaction of weather changes and malaria incidence is complicated, a likely explanation for such a low incidence, in addition to the intensive intervention of the research project, could be the atypical weather during the study period. During the years 2015 and 2016, the study area was seriously stricken by drought which was related to an *El Nino* event. Meteorological data from the study area show that rainfall decreased from 909 mm to 673 mm during 2011–2014 to 471 mm in 2015. At the same time, the average annual high temperature in 2015 (29 °C) was elevated with 2 °C compared to 2014 (27 °C).

Furthermore, the trial finding unexpectedly showed that the incremental protective effectiveness of either the combined intervention or the singleton intervention was not significantly different from the routine practice. While this result has been observed also in a few other studies [17], a majority of the empirical literature concludes that LLIN and IRS have substantial protective-effectiveness against malaria [17]. While we believe the internal validity of these results are good for the timing and context of this trial, the generalizability of the effectiveness and cost-effectiveness of combined implementation of LLIN and IRS are more uncertain.

We therefore performed a literature-based scenario analysis of cost-effectiveness for two reasons. First, we wanted to reduce a limitation of the trial-based evaluation—poor external validity. Second, we wanted to give decision-makers more flexibility to interpret results subject to a broader set of contexts.

In the literature-based cost-effectiveness analysis, we changed the input values from the trial-based analysis for malaria risk in the area (annual malaria probability) and protective-effectiveness of the interventions. We defined annual malaria probability as the probability of acquiring a malaria episode per person within a given year. We applied the annual parasitic incidence (API) measured as malaria probability per annum per 1000 population at risk. The API is the most common and reliable estimate of malaria probability in a specified geographic area [45]. In Ethiopia, about 17% of the districts on average have API lower than 5; and 43% of the districts have 5–100 API while nearly 7% has API greater than 100 [46]. Nearly 33% of the district are malaria-free. Based on the World Health Observatory data, the average API for Ethiopia is 58 per 1000 population at risk in 2015, And therefore we assume a base-case annual malaria probability of 5.8% (i.e. background malaria risk in the area) [44]. For intervention arms, we multiply the annual malaria probability by the protective-effectiveness of the interventions to estimate the transition probability in the corresponding arms with the presence of the interventions [34].

Regarding effectiveness, we utilised a systematic review to assume that the most likely values for protective effectiveness are 40% (35–45%) for LLINs alone and 28.5% (23.5–33.5%) for IRS alone [17]. For the combined intervention, we calculated protective-effectiveness as the multiplicative combination of the individual risk of malaria of the singleton interventions (LLIN and IRS) [47], yielding a protective-effectiveness of 57%.

### One-way sensitivity analysis

To test the robustness of model conclusion to these assumptions, we performed one-way sensitivity analyses on the literature-based cost-effectiveness model, in addition to the PSA. We did this for different level of protective-effectiveness of the combined interventions (47.1–67.1%) and at different level of annual malaria incidence (1–20%), and present results in a tornado diagram, where also the variables time horizon, cost, proportion of cases diagnosed, proportions of cases treated, probability of mortality from severe malaria were included. We evaluated the incremental cost-effectiveness values against the willingness to pay thresholds of less than or equal to 1 times GDP per capita.

## Results

The results of this paper are organized and presented in three parts. First, we describe the cost of the interventions and cost of malaria diagnosis and treatment from the providers’ perspective. Second, we present the cost-effectiveness analysis results based on trial based effectiveness and incidence estimates and cost data from the adjunct costing study, and together with probabilistic sensitivity analysis findings. Third, we present results from the literature-based cost-effectiveness analyses with probabilistic sensitivity- and one-way sensitivity analyses.

### Cost of interventions

The economic costs of malaria prevention interventions from the providers’ perspective are presented in Table 3. About 7740 LLINs were distributed with 99% coverage within LLIN arm and combination arm for about 3000 households. The annualized total cost for LLIN arm per 10,000 population was USD 10,641. About 88% of the cost is due to the bed net (LLIN) itself, while only 12% was expenditure for the delivery of the intervention (6% for personnel and 5% for transportation costs). Therefore, the unit cost of LLIN per person year-protected was USD 1.06. Similarly, with 95% of households covered with IRS costs a total of USD 30,660 per 10,000 population. From the total cost, about 58% (17,799) was spent for the purchase of the insecticide, and 26% (7883) was for personnel. The unit cost of malaria prevention with IRS alone per person-year protected was USD 3.07.

**Table 3:** Itemised cost of malaria prevention intervention per 10,000 population and unit costs in Adami Tullu, Ethiopia, 2014 USD

Costs and denominators	LLIN (% share)	IRS (% share)	LLIN + IRS (% share)
Costs of interventions per 10,000 population			
Personnel cost	675 (6)	7883 (26)	8216 (20)
The bed net cost	9321 (88)	NA	9058 (22)
The insecticide cost	NA	17,799 (58)	17,799 (44)
Materials and supplies	74 (0.7)	2232 (7)	2248 (6)
Transport costs	527 (5)	1561 (5)	1876 (5)
Training hall	44 (0.3)	1186 (4)	1211 (3)
Annualised total cost	10,641	30,660	40,408
Unit costs^a^			
Cost per person year protected	1.06	3.07	4.04
Cost per under-five child year protected	6.98	20.12	26.51
Cost per pregnant woman year protected	78.67	227.67	298.74
Cost per household covered	5.49	15.56	20.51
Total number of household in the study arms	1388	1527	1618

^a^The unit costs were computed by dividing the ‘annualized total cost’— which was incurred to implement the interventions—with corresponding denominator population. The denominators were drawn from the baseline survey

In the combined implementation of the interventions (LLIN + IRS), USD 40,408 was incurred in order to universally cover about 10,000 population with both LLIN and IRS. In the combined implementation, about 48%, 22%, and 17% of the cost was attributed to the cost of the insecticide (*Propoxur*), the personnel, and the bed nets (LLINs), respectively. The unit cost of combined intervention (LLIN + IRS) per person-year protected was USD 4.04 (Table 3).

Unlike the above three intervention arms, in the routine arm of the study, prevention intervention was implemented neither by the research project nor by the district health office. Therefore, the only cost incurred in this arm, from the health provider’s perspective, was the cost of diagnosis (testing) and treatment of malaria cases. The health systems provider’s perspective cost of diagnosis and treatment of malaria is presented in Table 4.

**Table 4:** Unit cost of diagnosis and treatment, and total cost per 10,000 malaria cases at primary health care units in Adami Tullu, Ethiopia, 2014 USD

Cost of diagnosis and treatment of malaria	Unit costs			Cost/10,000 cases
	Health centre	Health post	Average	
Diagnosis				
Personnel	0.12	0.41	0.26	2600
Materials and supplies	0.33	0.16	0.25	2500
Total cost of diagnosis	0.45	0.57	0.51	5100
Treatment				
Personnel	0.09	0.14	0.12	1200
Drug	1.06	1.06	1.06	10,600
Total cost of treatment	1.15	1.19	1.18	11,700
Total cost of diagnosis and treatment	1.60	1.76	1.69	16,800

### Trial-based cost-effectiveness results

The trial-based cost-effectiveness results (Table 5) indicate that the routine practice was not less effective than the other three alternatives, and therefore strongly dominates them because of lower costs. The expected costs from the model were 0.45, 22.16, 63.28, and 83.12 for routine practice, LLIN alone, IRS alone, and combined interventions, respectively. Combination (LLIN + IRS) was about 25% more costly than IRS alone and about four times higher than LLIN alone. In terms of expected health effectiveness, all the four alternative interventions averted almost similar amount of DALYs in a range of 10.26–10.27 DALYs (Table 5).

**Table 5:** Trial-based cost-effectiveness analysis ICER results, Adami Tullu, Ethiopia

Strategy	Cost (USD)	Incr cost	Eff (DALYs)	Incr eff (DALYs averted)	ICER
Excluding dominated					
Routine practice	0.5		10.26		
All					
Routine practice	0.5	0	10.259		0
LLIN alone	22.2	21.7	10.264	− 0.005	− 4528
IRS alone	63.3	62.8	10.266	− 0.007	− 9610
LLIN + IRS	83.1	82.7	10.265	− 0.006	− 13,546

**Fig. 2:**
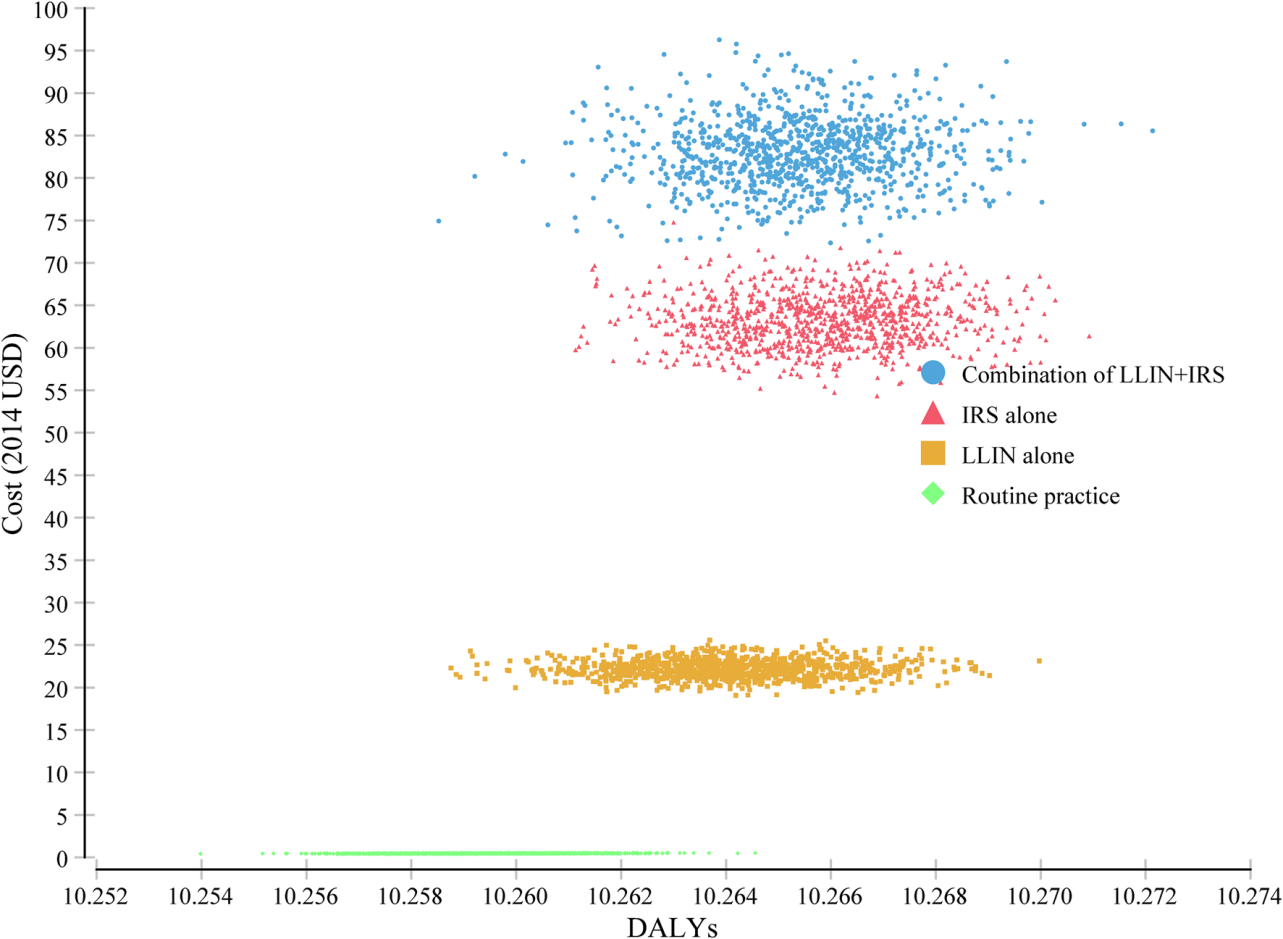
Scatterplot of the costs and health-effects of the four malaria prevention alternatives from the Monte Carlo Simulation (100,000 replication)

The probabilistic sensitivity analysis—the cost-effectiveness scatterplot for the four alternative malaria prevention strategy (Fig. 2)—indicates that the expected cost of LLIN alone had less variation and clearly lower than the cost of IRS alone

### Literature-based cost-effectiveness results

The literature-based cost-effectiveness analysis results are presented in Table 6. With the modified assumptions of intervention effectiveness and malaria incidence, the expected costs from the model were 1.87, 22.79, 64.09, and 83.41 for routine practice, LLIN alone, IRS alone, and the combined interventions, respectively. The combination intervention was almost one-third more costly than the expected cost of IRS alone (64.09), and about 3.5 times higher than LLIN alone. In terms of health-effect, the routine practice has the highest expected DALYs, while the combination of LLIN + IRS averted most DALYs and was the most effective of the three active alternatives. LLIN averted slightly more DALYs than IRS.

**Table 6:** Literature-based cost-effectiveness analysis ICER results, Adami Tullu, Ethiopia

Strategy	Cost (USD)	Incr cost	Eff (DALYs)	Incr eff (DALYs averted)	ICER
Excluding dominated					
Routine practice	1.9		10.451		
LLIN alone	22.8	20.9	10.350	0.101	207
LLIN + IRS	83.4	60.6	10.307	0.043	1403
All					
Routine practice	1.9		10.451		
LLIN alone	22.8	20.4	10.350	0.101	207
IRS alone	64.1	41.4	10.379	− 0.029	− 1422
LLIN + IRS	83.4	60.4	10.301	0.043	1403

IRS alone was ‘absolutely dominated’ by LLIN alone (i.e. IRS alone being more costly but less effective compared to LLIN alone). IRS alone was therefore eliminated from further consideration. The model predicts that the ICER for combination (LLIN + IRS) was USD 1403 per DALY averted compared to LLIN alone, and the ICER for LLIN alone was USD 207 per DALY averted compared to the routine practice.

**Fig. 3:**
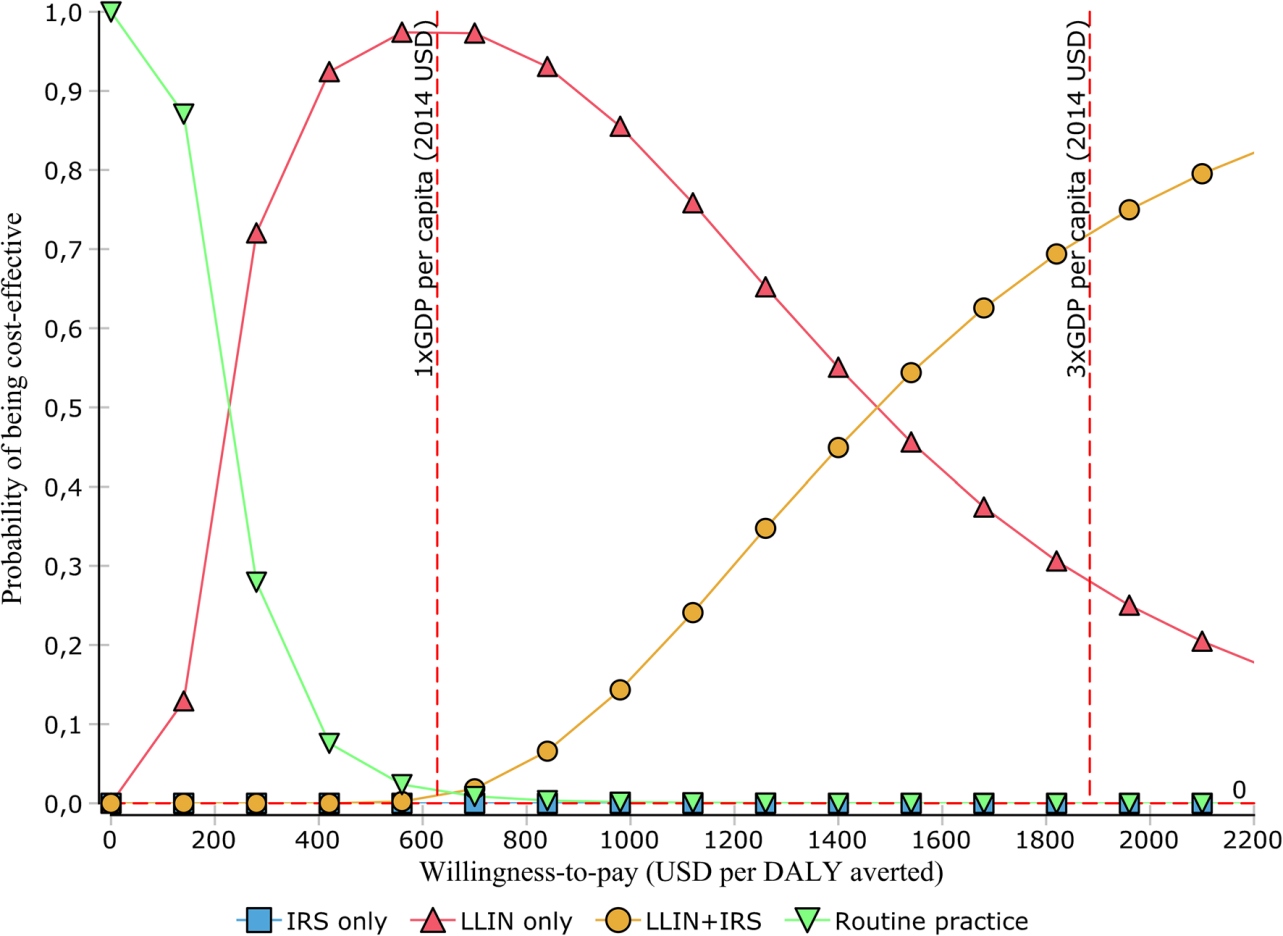
Cost-effectiveness acceptability frontier

Figure 3 shows the cost-effectiveness acceptability curves (CEAC) for literature-based CEA of the four malaria prevention alternatives at different levels of willingness to pay per DALY averted. For example, the probability of combined intervention (LLIN + IRS) being cost-effective option was less than 5% at a willingness to pay threshold of USD 628 per DALY averted while at a willingness to pay threshold of USD 1884 per DALY averted (3 times GDP per capita) the probability of the combined intervention being cost-effective was about 70%.

### Scenario analysis results of the literature-based model

#### Annual malaria probability

**Fig. 4:**
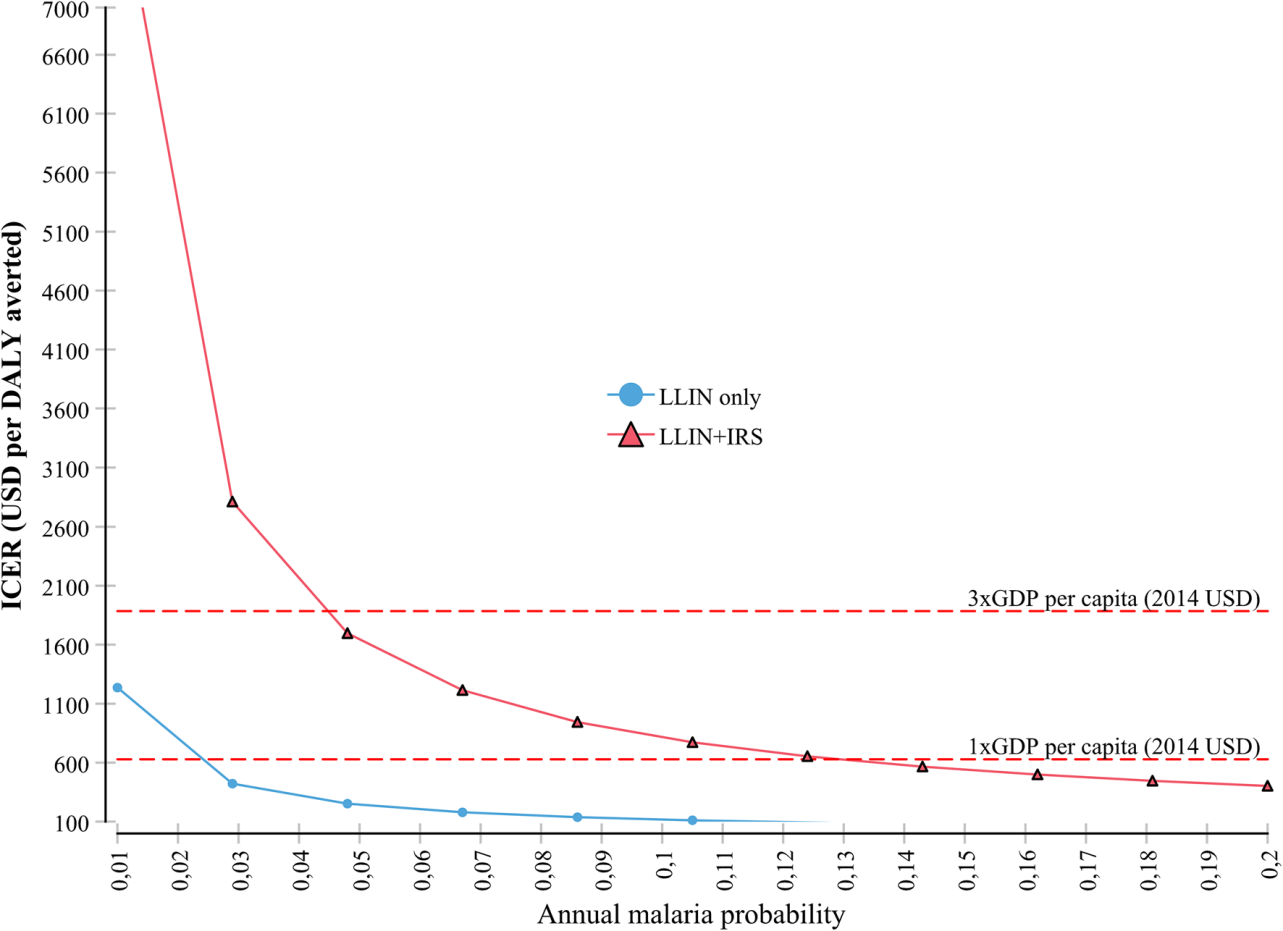
Sensitivity of ICER to variations in the annual malaria incidence in the area

In a one-way sensitivity analysis, we tested the effect of the background malaria risk on the cost-effectiveness of the interventions by varying the annual malaria probability from 1 to 20% while keeping all other variables at their base-case values. The results show that the combined intervention (LLIN + IRS) becomes cost-effective compared to LLIN alone when the annual malaria incidence is higher than about 13% if the WTP threshold is defined at 1 times GDP per capita per DALY averted. The LLIN alone becomes cost-effective compared to the null intervention when the malaria incidence is higher than about 2% per year (Fig. 4). If we defined the willingness to pay threshold at 3 times GDP per capita, the combined intervention becomes cost-effective in areas where the annual malaria probability is higher than 4.5%.

#### Protective-effectiveness of the combined intervention

**Fig. 5:**
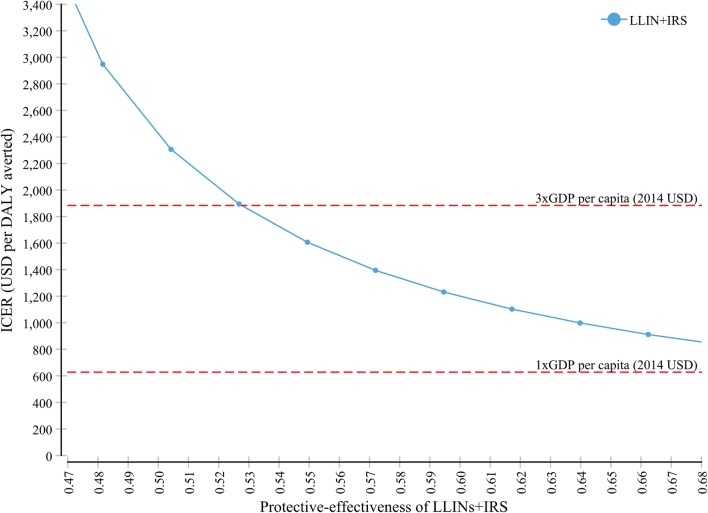
Sensitivity of ICER to variations in the protective-effectiveness of combined intervention

At 5.8% annual malaria probability, the ICER of the combined intervention is not likely to be lower than GDP per capita when its protective effectiveness is varied ± 10% (47.1–67.1%), given that the IRS alone reduced the malaria probability by 28.5% and LLIN alone by 40%. The protective-effectiveness of combined implementation (LLIN + IRS) should be higher than 53% in order for the ICER to be in a range of 3 times GDP per capita per DALY averted (Fig. 5).

**Fig. 6:**
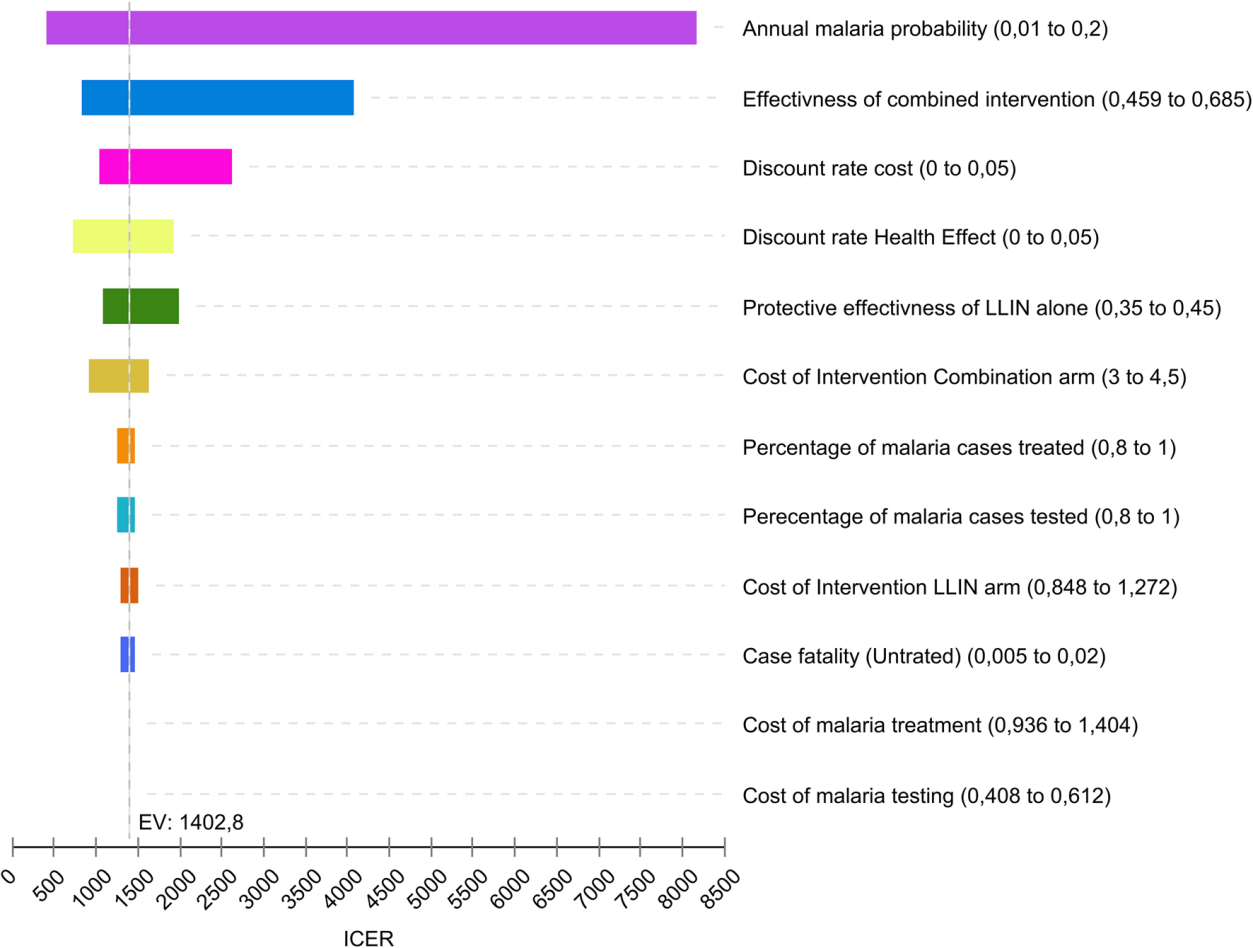
Tornado diagram—ICER LLIN + IRS vs. LLIN only

Similarly, one-way sensitivity analysis with the Tornado diagrams shows that the ICER of the combined intervention in the literature-based analysis was mainly sensitive to change in annual malaria incidence in the area and the level of protective-effectiveness of combined intervention (Fig. 6). In addition, variability in the discount rate of costs and health-effect, and protective-effectiveness of LLIN alone modestly influenced the ICER.

## Discussion

This cluster randomised controlled trial found no significant difference in the effects of malaria prevention. The effectiveness of all the three intervention arms was the same as the routine arm, and the economic evaluation inevitably shows that the current routine practice dominates all the prevention alternatives since they are all more costly. When generalising key inputs from the trial and replacing them with literature-based assumptions, the economic evaluation shows that both—LLINs alone with ICER of USD 207 and the combined intervention with ICER of USD 1403—are likely to be ‘cost-effective’ compared to a willingness to pay threshold of 3 times GDP per capita per DALY averted. At a willingness to pay threshold of 1 times GDP per capita, only LLIN alone is likely to be cost-effective, while IRS is dominated by LLIN (more costly but less effective).

This study is the first of its kind in Ethiopia which compared the cost-effectiveness of malaria prevention interventions. Our literature-based analysis yield higher ICERs for both the combined intervention and LLIN alone compared to previous studies on malaria prevention [7]. For example, Goodman et al. analysing the cost-effectiveness of malaria in sub-Saharan Africa, found an ICER (in 1995 USD per DALY averted) ranging only from 32 to 58 for ITN and from 16 to 29 for IRS [48]. Another study by Morel et al. [49] examined the cost-effectiveness of mixes of curative and preventive interventions, and reported an ICER (in 2005 international dollar per DALY averted) ranging from 10 for case management with artemisinin-based combination therapy to 96 for combination of the four interventions together (i.e. IRS, insecticide-treated net (ITN), case management with artemisinin-based combination therapy (ACT), and intermittent presumptive treatment in pregnancy). A systematic review of studies published between 2000 and 2010 [6] reported a median ICER of 27 per DALY (range 8.15–110) for LLIN/ITN and 143 per DALY (range 135–150) for IRS.

The relatively high ICERs in this study compared to other studies in Africa can be partly explained by the differences in malaria burden, the incremental costs of interventions, and unique malaria dynamics in Ethiopia. In the last 15 years, the incidence of malaria in sub-Saharan Africa decreased significantly [2, 44, 50], while the cost of the interventions increased [51]. The cost of the intervention increased mainly because of the replacement of DDT (Dichlorodiphenyltrichloroethane) with Propoxur, use of LLINs instead of ITNs, and the introduction of the ACT. All these three recent changes were not only associated with improving malaria prevention and control, but also with increased cost to the health system. Particularly IRS was costly in our study and this was mainly caused by the price of the insecticide. In theory, although insecticide resistance can be one of the factors which can affect the cost-effectiveness of IRS or combined intervention, it is less likely that it’s’ effects could influence the results in our trial. Our entomological study indicates that the efficacy of the insecticide (Propoxur) applied in all the study arms was similar and very potent in all study arms for the whole year [MalTrials Final report, Unpublished]. In addition, the difference in malaria epidemiology in Ethiopia compared with other places in African or elsewhere could also largely contribute to this disparity [52]. The epidemiologic profile of malaria in Ethiopia is in a number of ways different compare to other African countries. For example, malaria transmission in Ethiopia is low to moderate, unstable, and seasonal while it is high, stable, and perineal elsewhere [52, 53].

Practically, economic evaluation of malaria prevention interventions is complex [7, 54]. Unlike typical cost-effectiveness evaluations, in some cases, the effects of combined interventions might be the same with the effect of individual interventions alone; and subsequently, the incremental effect could be negligible. In other cases, any of the intervention might not be effective at all—even compared with ‘doing-nothing’ [17]. In our study also we found that the effectiveness of the combined intervention was the same with both singleton and routine interventions. This might be partly explained by the ‘counter-balanced effect’ between incremental health effect and cost saved resulting from adding IRS over high LLIN coverage or vice versa. On the other hand, the strong protective effect from active case finding and treatment by itself might dilute the ‘modest’ protective-effects from other preventive measures (i.e. LLIN and IRS). It is also important to note that in this trial—across all the four study arms—a weekly visit to each household was conducted in order to identify any febrile member of the household, and almost all febrile cases were tested with RDT, and if found positive, treated with the appropriate ant-malaria drug [28]. Therefore, we strongly recommend further pragmatic trials from different setting from our study to estimate protective-effectiveness of the intervention.

In general, the cost-effectiveness of malaria prevention intervention is a function of the health benefit gained and the resources required to implement the intervention [27]. In the one-way sensitivity analysis, first, we tested the effect of the background malaria incidence in the area on the cost-effectiveness of the interventions by varying the annual probability of malaria infection for an individual from 1 to 20%. On account of this, the ICER for combined intervention varied from about USD 8000 to USD 200 per DALY averted. Moreover, the annual malaria incidence should be at least about 13% in order for the combined intervention to be cost-effective compared with a willingness to pay threshold of 1 times GDP per capita per DALY averted for Ethiopia (USD 628). However, what the recent data from Ethiopian Ministry of Health indicates is that only a few areas in Ethiopia have malaria risk levels of such magnitude. Only about 5% of the districts in Ethiopia, mostly in Western lowlands and few in the Rift Valley, have annual incidence rates exceeding 13% [46], and based on the results of this analysis should be the focus of attention for future prevention campaigns.

These findings should be interpreted in the light of at least two important issues about the dynamics of malaria control program at low incidence setting (i.e. at stages of elimination and eradication) should look like. First, malaria control program should not be a victim of its own success [55]. When the malaria control program succeeds, malaria incidence will certainly reduce. In this case, such a versatile malaria prevention interventions like IRS and LLIN will not continue to be sufficiently competitive in terms of cost-effectiveness parameter, and LLINs and IRS will both appear to be not cost-effective [55]. Therefore, it has been argued that for malaria prevention programs the willingness to pay thresholds should be expanded from the conventional level [56]. Second, the need for disaggregate malaria data at a district level is crucial for better targeting of interventions and for local planning (micro-planning). In this regard, the National Malaria Control Program in Ethiopia has also recently stratified all districts based on annual malaria incidence into four groups (i.e. free, low, moderate, and high) and started conducting interventions based on the strata [46].

The second parameter that we examined in the one-way sensitivity analysis was the protective-effectiveness of combined intervention. We found that the combined intervention (LLIN + IRS) need a minimum of 53% protective-effectiveness in order to be ‘cost-effective’ alternative (Fig. 5). It is important to remember that none of the interventions have an inherent degree of effectiveness. Rather, it is the manner how it is implemented, the identification of those areas where it is most suitable, and the proper use by the community which determine the effectiveness most. However, based on a recent systematic review [17], it would be very challenging to achieve a protective-effectiveness of such level (53%) against the current supply side and demand-side barrier which reduces the effectiveness of both individual and combined interventions. The major demand side barriers for LLIN, observed in our visits, includes under-utilization, misuse, and lack of convenient sleeping space to hang-up the bed nets; while refusal, covering the wall of the house with a mud or other material, and rudimentary nature of the wall for some of the houses were challenges for IRS. The financial and human capacity of the district to execute the interventions, the price of the insecticide, and the quality of the LLINs can be considered as major supply-side barriers. IRS demands strong and very close supervision.

The costing analysis shows that the unit cost of IRS per person-year protected was predominantly influenced by the price of the insecticide, which alone accounted for about sixty percent of the cost. Regarding the cost of LLIN, in addition to the price of the bed nets, useful life-year (durability) of the bed nets was important parameters which determined the cost of LLIN per person-year protected. The life-year of the LLINs determines the frequency of the redistribution (refill). In Ethiopia, based on the National Malaria Program, LLINs were intended to serve for about 3 years and therefore the distribution campaigns are held once every 3 year [11]. However, what we observed in our study was that the LLINs worn out faster, and had little effect after 1 to 2 years. Local production of the bed nets with low cost and better quality could reduce the price of the bed nets. A strong quality control mechanism in the production, procurement, and distribution of the nets can be considered not only to maintain the fabric integrity of the nets but also to maintain the insecticidal property. Above all, a well-coordinated information, education, communication and advocacy program promoting proper utilization of the LLIN could improve both effectiveness and longevity of the LLINs.

In this study, most of the cost items of malaria prevention interventions at the district level were identified, measured, and valued prospectively alongside the community trial using robust techniques [27]. Yet, there are some caveats that deserve due consideration with respect to the data, generalizability, and relevance of this study. The first limitation was that the costing was done only from the local providers’ perspective and a few cost items incurred at national and regional levels (e.g. mass-media and communication costs etc.) were omitted. Although this might not have substantial implication when we compare the cost and cost-effectiveness of the prevention interventions, this might to some extent underestimate the actual unit cost of the interventions.

The second limitation to our model was that we were not able to account for health-loss from co-morbidities of severe malaria such as anaemia, convulsions, and long-term neurological sequel because of lack of accurate estimates about the magnitude of these events. Despite the effect on the ICER would be minimal since the probability of severe cases is rare in the cases of treated malaria [36], this might underestimate the actual benefit of the prevention intervention slightly [57].

A third limitation of this study is in the decision we made in choosing 1 times or 3 times GDP per capita per DALY averted as a willingness to pay thresholds for interpretation of the ICER results. Despite long-standing debate in economic evaluation literature on this issue [58], it is particularly important for the evaluation of malaria prevention interventions in Ethiopia [57]. It is difficult to precisely define the WTP threshold in Ethiopia due to the fact that the financing of health care in general and malaria programs, in particular, are complex. For example, the larger share of the funding (78.6%) for malaria is generated from different to external sources (UNICEF, PMI, GLOBAL FUND, WHO etc.) and most of which is also ear-marked for malaria (vertical program) [59]. This kinds of cost-effectiveness evidence would be most relevant in a country where there is functional and established disease control priority-setting system which utilizes economic evaluation in decision making [60].

## Conclusions

Based on the current trial-based analysis, LLINs and IRS are not cost-effective compared to routine practice. However, based on the literature-based analysis, LLIN alone appear as likely to be cost-effective if willingness to pay is defined at 1 times GDP per capita per DALY averted, while IRS is dominated by LLIN (i.e. more costly but less effective). The annual malaria risk in the area and protective-effectiveness of combined intervention and LLIN are the key determinants of the cost-effectiveness of the interventions. Malaria program implementation should provide high focus to the improvement of the protective-effectiveness of IRS and LLIN.

## Abbreviations

ACT: artemisinin-based combination therapy
CEA: cost-effectiveness analysis
CEAC: cost-effectiveness acceptability curves
DALY: disability adjusted life year
ETB: Ethiopian Birr
GDP: gross domestic product
ICER: incremental cost effectiveness ratio
IRS: indoor residual spraying
LLIN: long lasting insecticidal nets
PMI: Presidential Malaria Initiative
RDT: rapid diagnosis test
USD: United States Dollar
WHO: World Health Organization
WTP: willingness to pay
